# Interventions Designed to Improve the Learning Environment in the Health Professions: A Scoping Review

**DOI:** 10.15694/mep.2018.0000211.1

**Published:** 2018-09-12

**Authors:** Larry Gruppen, David M. Irby, Steven J. Durning, Lauren A. Maggio

**Affiliations:** 1 University of Michigan Medical School; 2 University of California; 3 Uniformed Services University

**Keywords:** learning environment, educational climate, systematic review, educational interventions, theory development

## Abstract

This article was migrated. The article was marked as recommended.

**Purpose:** To identify and describe interventions designed to affect the learning environment (LE) in health professions education, summarize factors that influence the LE, and determine gaps that require additional research. The LE can be thought of as a dynamic and complex construct co-created by people in a particular setting. A positive LE represents a welcoming climate for learning, which enhances satisfaction, well-being, academic performance and collaboration, while a negative LE restricts participation and learning, leading to emotional exhaustion, depersonalization and burnout.

**Method:** A six-step scoping review methodology was followed to identify and report on literature that describes interventions affecting the LE in the health professions education: 1. Identify the research question, 2. Identify relevant studies, 3. Select studies to be included, 4. Chart the data, 5. Collate, summarize and report results, and 6. Consult with stakeholders.

**Results:** 2,201 unique citations were identified and reviewed using titles and abstracts. 240 full-text articles were retained for detailed review, resulting in the inclusion of 68 articles. Study results are reported in relation to essential components of the LE: personal, social, organizational, physical and virtual spaces. Results of four different types to the studies of the LE are described: specific *interventions*impacting the LE, *comparisons*of perceptions of the LE by two or more different groups, *associations with other variable* such as well-being with the LE, and *descriptive*studies of the LE. Major influences included accreditation regulations, curricular interventions, faculty/staff development grading practices, instructional interventions, placements, physical and virtual spaces, and support services; and are reported along with specific interventions.

**Conclusion:** These results reflect the complexity of the LE and the need for conceptual clarity. Since the quality of the evidence was not evaluated, the identified influences should be viewed as potential opportunities to improve the LE.

## Introduction and Purpose

Educational learning environments (LE) dramatically affect the way participants think and feel, engage and work. Positive LEs support learning and are welcoming, collaborative, ( Chinthammitr and Chierakul, 2014; Thomson *et al.*, 2014; Tackett *et al.*, 2017) and respectful while negative or “chilly” LEs ( Janz and Pyke, 2000) are destructive and restrict participation and learning. LEs describe the dynamic, co-constructed perceptions, experiences and behaviors of participants in the physical and virtual spaces within which learning occurs. But more importantly, it also refers to the tone of the educational climate or culture, and the routine way people interact. LEs affect a wide variety of factors important to learners and providers alike: burnout, depersonalization and emotional exhaustion; satisfaction and well-being; identity formation; performance and collaboration ( Darcy A. Reed *et al.*, 2011; Thomson *et al.*, 2014; Castillo-Angeles *et al.*, 2017; Tackett *et al.*, 2017). While interventions designed to improve LEs for health professionals have targeted many of these factors, which interventions have been studied? Given the diversity of ways LEs have been defined, how can these interventions be identified and categorized? If we could find such interventions, we could better target efforts to improve the learning environment for all. The purpose of this scoping review is to identify and classify interventions designed to improve the environment for learning in the health professions.

By interventions, we mean the introduction of a planned new activity (e.g., near peer coaches) or organizational change (e.g., curriculum, training site, duty hours) that is anticipated to have an impact on the learning environment. Our primary purpose in this review is to identify interventions that could improve the LE, but we also recognize that it is important to understand the factors that influence the LE, whether included in formal interventions or not. Thus, we cast our net more broadly than just a focus on interventions *per se.*

The learning environment (LE), which appears frequently in the health professions education literature, is a complex theoretical construct that lacks a unified definition ( Genn, 2001b, 2001a; Roff and McAleer, Sean Sue Roff, 2001). The conceptual ambiguity surrounding this term has arisen, in part, from the varying disciplines and associated theoretical lenses used to investigate this phenomenon (i.e., anthropology, education, psychology, and sociology). The LE can describe personal experiences and perceptions (psychology and education), social interactions (sociology and education), organizational culture and practice (anthropology and sociology), physical facilities and online spaces (sociology and education) within which learning occurs. It can be associated with formal and informal learning experiences that occur in classroom, online, simulation and clinical settings.

The LE is often used interchangeably with such terms as *atmosphere*, *educational environment*, *learning climate*and *organizational culture.* The LE has been defined as “a set of features that gives each circumstance and institution a personality, a spirit, a culture and describes what it is like to be a learner within that organization” (p. 553) ( Holt and Roff, 2004). However, just what these features are is inconsistent from one situation to another and from one study to another. The LE can be thought of as a complex psycho-social-physical construct co-created by individuals, groups, and organizations in a particular setting, and shaped by contextual climate and culture ( Palmgren, 2016).

There is little disagreement that the LE is important, linked to various educational outcomes ( Genn, 2001a, 2001b), and the focus of a number of accreditation regulations (e.g., LCME, ACGME, GMC). While the perceived importance of the LE has led to numerous efforts to measure it ( Colbert-Getz *et al.*, 2014), there is still a lack of clearly identified, evidence-based interventions or conditions that positively impact the environment for learning in the health professions.

### Conceptual framework for the learning environment

Although many authors do not provide an explicit theoretical perspective on the LE in their studies, we believe that the LE can be best understood and studied through the lens of sociocultural learning theories that include situated cognition, situated learning, ecological psychology, and workplace learning. The LE is conceived by different people in different ways, is dynamic and emergent, and is co-constructed through interactions and activity. Within the situated learning framework, learning involves acculturation into a new knowledge community or community of practice through active participation - initially as a legitimate peripheral participant and emerging into a full participant ( Lave and Wenger, 1991). Ecological psychology and workplace learning emphasize that social interaction is facilitated through affordances in the learning/working environment (tools, scaffolded relationships, tasks, language, concepts) and the active engagement of learners (through their agency, engagement and emerging autonomy) ( Billett, 2001). Situated cognition theorizes that learning is social and involves an interaction between persons and environment - thus linking learning, situations and culture. Specifically, knowledge is embedded in the activity, context and culture in which it is learned ( Brown *et al.*, 1989).

Each of these theories emphasizes the importance of interactions and collaborations with others “as the means for students’ learning/participation, both through learning knowledge and skills from others, and through becoming familiar with the norms, cultural beliefs and attitudes existing in the communities to which they (the learners) are being introduced” (p. 739) ( Schönrock-Adema *et al.*, 2012). However, the LE construct extends beyond typical sociocultural frameworks to include intra-individual psychological characteristics (learning preferences and history), as well as institutional culture, organizational structures and physical and virtual spaces in which students learn. It should be emphasized that the LE is not “owned” by any particular theoretical perspective. Neither is the LE often a central concern, which leaves the construct in something of a theoretical limbo.

### Components of the learning environment

**Figure 1.**
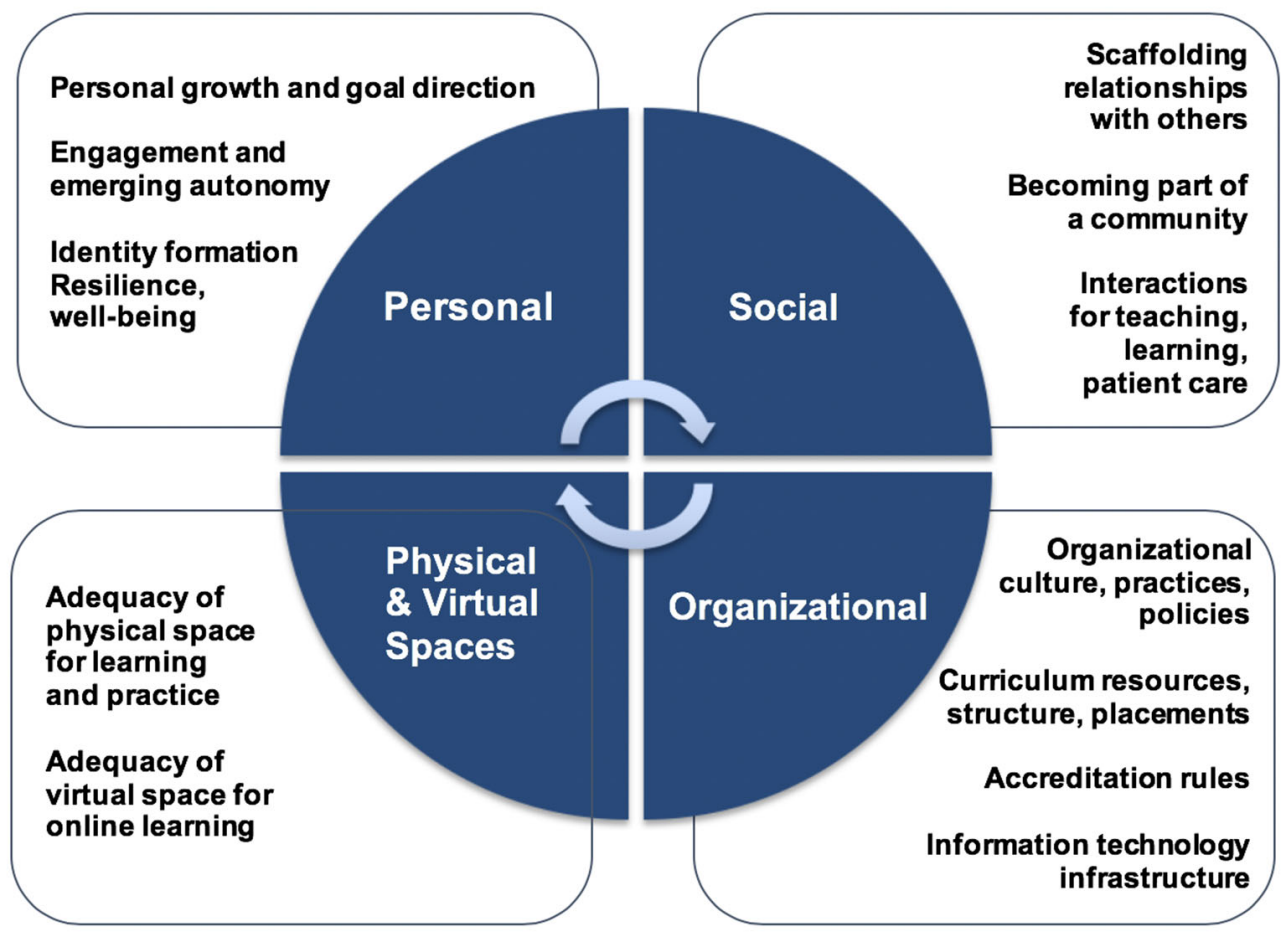
Four interactive components of the learning environment: personal, social, organizational, physical and virtual.

Lacking a canonical theory of the LE from the health professions education literature, we sought to synthesize multiple conceptual frameworks ( Moos, 1974, 1980; Genn, 2001b; Schönrock-Adema *et al.*, 2012; Gruppen *et al.*, 2015; Gruppen and Stansfield, 2016; Gruppen, Rytting and Marti, 2017) and identified four overlapping and interactive core components ( Figure 1):

1. *Personal Component.* The individual learner interacts with the LE through activity, develops perceptions of the LE, and engages in personal growth through clarity about goals, selection of relevant and meaningful learning; and in the process develops professional identity and increasing autonomy.
2. *Social Component.*Learners engage with others and navigate multiple relationships, which shapes their perceptions of and experiences with the LE. These relationships include: peer-to-peer (competition, cooperation, shared values and learner culture), learner-to-faculty/staff (trust, feedback, communication, instructional strategies, mentoring), and learner-to-patient (responsibility, acceptance and trust). All of these social relationships influence what and how students learn.
3. *Organizational Component.*Organizations provide structure, guidance and support for learning, including curriculum resources and artifacts, geographic placements, accreditation rules as well as organizational practices, culture and policies (orderly environment, rule clarity, duty hours, regulatory environment, teacher control, curriculum, placements, technology infrastructure). One example of this is the Clinical Learning Environment Review (CLER) implemented by ACGME. The underlying premise of the CLER program is that the educational program and patient care will be improved if constructive actions are taken regarding patient safety, health care quality, care transitions, supervision, fatigue management, and professionalism (Accreditation Council on Graduate Medical Education, no date; Weiss, Wagner and Nasca, 2012). We also include placements in the community (geographical settings/locations) in this organizational component as well.
4. *Physical and Virtual Component.*Learning and practice take place within physical spaces of educational and practice settings. Similarly, informational infrastructures and resources (e.g., online resources, electronic health records) also provide a virtual “space” in which learning is fostered or obstructed.

These components serve as an organizing framework for the diverse and often implicit definitions of LE for this review, but they do not constitute a complete theory of the learning environment in the health professions education. Such a theory will require considerable debate and discussion within the community. Nor are our categorization of individual studies definitive; most studies include elements from more than one component.

### Studies of the learning environment

We conducted a scoping review of the literature to identify and characterize interventions that appear to affect the LE in order to better prepare health professionals for delivering quality patient care and engaging in a fulfilling practice. Recognizing that different phases of training are done in very different LEs, this review includes pre-clinical, clinical, simulation, and online LEs. The research questions are:

• What interventions affect the LE in the health professions?
• What components of the LE are targeted by these interventions? Which are ignored?
• What are the theoretical and practice gaps that require additional research on LE interventions?

## Methods

We chose a scoping review to determine the extent of the literature on LE interventions and associated factors, which our preliminary search indicated might not be extensive enough for a full systematic review of the literature. Additionally, we did not set out to evaluate the efficacy of the influences, but rather to characterize for the health professions education community the types of interventions used to improve the LE. To guide this scoping review, we utilized Levac’s ( Levac, Colquhoun and O’Brien, 2010) modified version of Arksey and O’Malley’s methodological framework ( Arksey and O’Malley, 2005) for scoping reviews. This framework includes six steps, which we used to organize our methods (Steps 1-3) and results (Steps 5-6).

### Step 1: Identify the research question

Based on several conference calls, we collectively discussed and agreed upon the purpose and rationale for this review, which informed the formulation of our research questions. In our discussions, we considered the population, types of relevant interventions, and impact on the LE.

### Step 2: Identify relevant studies

We assembled a research team with expertise in health professions education, clinical medicine, and information science. All team members had interest and experience in health professional LEs as well as experience in conducting literature reviews in health professions education.

LM, a health professions education researcher trained in information science, collaborated with a medical librarian to search and manage results from PubMed, Embase, Scopus, CINAHL and ERIC. With input from the team, search strategies were crafted using Boolean operators to combine controlled vocabulary terms (e.g., medical subject headings) and key words for all relevant concepts (search details available in Appendix 1). Our searches, were carried out beginning in August 2017 and were finalized 11 October 2017. The searches focused on journal articles written in English. No date limits were set and both quantitative and qualitative studies were included.

### Step 3: Select studies to be included in the review

The research team collaboratively determined inclusion criteria based on our research questions. For inclusion, articles needed to describe a study of an educational intervention or associated factor that measured outcomes related to the learning environment and that targeted health professions trainees and/or practitioners. Therefore, we excluded articles only focused on measuring the LE and/or that did not include a clearly identified intervention on the LE.

Our initial study selection, based on titles and abstracts, was an iterative process conducted over regular phone meetings. To ensure concordance on the inclusion criteria, we participated in several rounds of selecting studies as a group. In total, each reviewer examined approximately 500 titles and abstracts. When moving to independent selection, we continued group discussions for any studies for which inclusion was uncertain. If consensus was unmet based on the title and abstract, the full-text was reviewed and consensus was achieved.

### Step 4: Chart the data

We collectively created a data charting form, which was adapted from a data extraction tool utilized by the Best Evidence Medical Education Collaboration for knowledge syntheses in health professions education ( Issenberg *et al.*, 2005) and tailored to our research questions. Before implementation, we tested the form on four citations as a group to ensure agreement. Upon agreement, we each independently charted data for approximately 50 articles with one of the authors (DI) reviewing an additional 40. Following data charting, we held weekly phone calls to pose questions and ensure consistency in how we extracted study information.

### Step 5: Collate, summarize and report results

Our database search identified 2,201 unique citations; 68 met the inclusion criteria. See results.

### Step 6: Undertake consultations with stakeholders

This will be completed at a conference convened by the Josiah Macy, Jr. Foundation in April 2018 to identify policy recommendations for improving the LE for the health professions.

## Results

**Figure 2.**
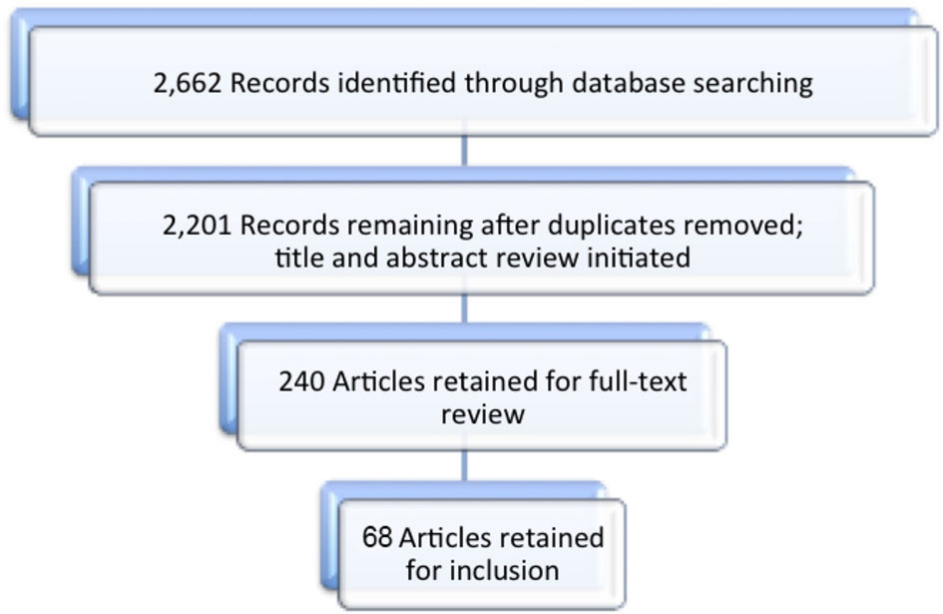
Review and selection of articles on learning environment interventions in health professions education.

Our search retrieved 2,662 articles (PubMed=1,491; CINAHL=77, ERIC=132, Scopus=244, Embase=718); with duplicates removed there were 2,201 unique citations. Based on examination of all titles and abstracts, 240 full-text articles were selected for review. Following full-text review, 68 articles were retained for inclusion ( Figure 2). In the set of studies, there were 18 nations represented and six professions studied (medicine n=54; nursing n=11; dentistry n=1, pharmacy n=1, veterinary n=1, chiropractic n=1). Preclinical and clinical students were the primary population (n=45), but several studies also included residents (n=12) and/or faculty members (n=4). In some cases, studies included more than one population, setting and/or profession.

We identified four approaches to the study of interventions in the LE. First, there are studies designed to assess the impact of a specific intervention or series of interventions on the LE. These include studies of changes in duty hours, curricula, placements, and faculty development and their impact on LE. These we term **interventional** studies. Second, investigators compared two different groups’ assessments of the LE related to instructional formats, curriculum models, geographical placements, and grading practices. We titled these **comparison group** studies. Third, one or more variables of interest, such as resilience, burnout, mistreatment, achievement, and well-being, were associated with perceptions of LE. We called these **association** studies. Fourth, descriptive studies using qualitativemethods illuminate participant perspectives and identify themes associated with interventions in the LE, such as establishing a welcoming environment and teaching culture, continuity of participants, and availability of learning/practice space. We termed these as **descriptive** studies. Each of these four approaches offer important insights into interventions impacting the LE.

The results of the review are organized around these four approaches to studying LE interventions.

### Interventional Studies

Sixteen studies described specific interventions to improve the LE ( Table 1). One study aimed at the personal component, three studies addressed the social component, and 12 examined the organizational component; none targeted the physical/virtual component. In the personal component, time spent by students on direct patient contact is positively related to their perceptions of the quality of the LE. In the social component, a formative assessment tool supported student’s clinical learning and improved perceptions of LE, and supervision by the same preceptor created a more supportive relationship.

The organizational interventions can be clustered into changes in duty hours (mixed response of impact on LE), curricula (preparation for clerkship program, teamwork skill training and mistreatment program improved LE), and faculty development (faculty development, train the trainers, and teaching skills workshops all improved LE).

**Table 1.** Themes from 16 studies of interventions to improve the learning environment in the health professions.

LE Components - Citation, Nation, Profession	Interventions	Findings (+, =, -)
Personal		
Van Hell (2009) ( Van Hell, Kuks and Cohen-Schotanus, 2009) The Netherlands Medicine	Students tracked their allocation of time to clerkship activities and perceptions of LE	(+) Student time spent on direct patient contact is positively related to their perceptions of the LE quality.
Social		
Cottingham (2008) ( Cottingham *et al.*, 2008) USA Medicine	Implemented a school-wide culture change project using appreciative inquiry and focused on everyday relational patterns	(+) Student satisfaction with educational experience rose sharply and reflective narratives described significant constructive change in the LE.
Engstrom (2017) ( Engstrom *et al.*, 2017) Norway Nursing	Introduced a formative assessment tool for students, preceptors and nurse teachers in mid-course and final assessments	(+) Assessment data supported students’ clinical learning, structured content of conversations, and improved perceptions of the LE.
Sundler (2014) ( Sundler *et al.*, 2014) Sweden Nursing	Paired nursing students with a personal preceptor throughout rotation or a nurse preceptor of the day	(+) Students with the same preceptor throughout were more positive about the supervisory relationship and pedagogical atmosphere.
Organizational		
Edafe (2013) ( Edafe, Mistry and Chan, 2013) UK Medicine	Introduced pre-clinical FAIRness (feedback, activity, individualization, relevance) teaching methods course in preparation for first clinical rotation	(+) FAIRness group students felt more integrated with the teams than control group students; and less impacted by lack of structure and demoralization than control group
Henderson (2010) ( Henderson *et al.*, 2010) Australia Nursing	Implemented a staff development program for capacity building in nursing	(+/-) Students rated the psycho-social LE higher during intervention than prior to or post intervention
Hunter (2004) ( Hunter *et al.*, 2004) USA Medicine	Utilization of hospitalist v. non-hospitalist teachers on inpatient medicine rotations	(=) No significant differences in LE, education time, teaching style, evaluation, feedback and contributing to student growth and development
Lachance (2014) ( Lachance *et al.*, 2014) Canada Medicine	Implemented 16-hour workday	(-) Surgical residents and professors perceived duty hour restrictions negatively impacted the LE; professors more so than residents
Lau (2017) ( Lau *et al.*, 2017) USA Medicine	Implementation of a surgical rotation specific mistreatment program	(+) Students reported improved understanding of mistreatment, increased opportunities to share experiences, and a more supportive environment. The number of mistreatment reports decreased annually following implementation
Moutier (2016) ( Moutier *et al.*, 2016) USA Medicine	Launched a multi-pronged institutional change campaign targeted at faculty to improve healthy, respectful learning environment	(+) Faculty reported declines in derogatory comments, anger outbursts, hostile email or speech post intervention, and diminished work productivity as a result of disruptive behaviors
Moystad (2014) ( Moystad *et al.*, 2015) Norway Dentistry	Implemented a faculty development program for clinical teachers	(+) Participants perceived improvement in LE, and increased collaboration and calibration among teachers
Nishioka (2014) ( Nishioka *et al.*, 2014) USA Nursing	Implemented a dedicated education units (DEU) for students	(+) Students perceived clinical learning experiences and mentoring relationships in DEUs more highly than students in traditional units
Rubak (2008) ( Rubak *et al.*, 2008) Denmark Medicine	Offered a 3-day train-the-trainers course for medicine and surgery faculty	(+) Participants reported an improved knowledge of teaching skills and perceptions of the LE compared to control group
Schumacher (2014) ( Schumacher *et al.*, 2014) USA Medicine	Implemented 2011 ACGME duty hours	(-) Over half of residents reported worsening care continuity, handoffs, and senior resident workload had worsened; four aspects unchanged, including supervision and quality of care. Most residents reported amount of sleep unchanged.
Spickard (1996) ( Spickard, Corbett Jr and Schorling, 1996) USA Medicine	Held 3-hour teaching skills workshops for residents designed to help participants provide feedback and create a constructive LE	(+, =) Student ratings of residents’ ability to create a constructive LE and provide feedback were higher for participants than non-participants; overall ratings of teaching unchanged.
Wallin (2015) ( Wallin *et al.*, 2015) Sweden Medicine and Nursing	Implementation of a 3-day education module for training surgical teams of specialist nursing students and residents in safe teamwork skills in an authentic operative theater	(+) Participants perceived the safety climate, teamwork climate and readiness for interprofessional learning more positively than conventional program participants
Physical and Virtual Spaces		
None identified		

### Comparison Group Studies

Comparison group studies were the most common of the four approaches and also quite diverse in how the comparison conditions were defined. Some were naturally occurring differences in the LE (e.g., in two different clinical sites) whereas others were side-effects of events or changes (e.g., institution of team-based learning). These 29 studies were sorted by personal component (1 study), social component (3 studies), organizational component (23 studies), and the physical/virtual component (2 studies). See Table 2. Within the personal component, nursing students with and without prior experience with elder care perceived the nursing home LE similarly. In the social component, distance learning compared with local live learning were perceived similarly, yet learners tended to prefer traditional classroom environments. Blended learning, the combining of online and in-person learning, was preferred to traditional instruction.

In the organizational component, geographical placements were compared (rural/remote preferred to metropolitan referral centers) and curriculum models contrasted (integrated and problem-based preferred to traditional discipline curriculum). Also, school features, the presence of learning communities and pass/fail grading practices effects on LE were explored. In terms of their effects on the LE, highly-rated departments had legitimacy, good clerkship arrangements, and a focus on personal development and engagement of learners; schools with learning communities had more positive student perceptions of LE than schools without learning communities; and students in schools with grades had higher stress, emotional exhaustion and depersonalization than students in pass-fail schools.

In the physical/virtual component, medical students had higher overall satisfaction than residents with Veterans Administration hospital training, although students’ satisfaction declined over time while residents improved. The LE for obstetrics and gynecology residents in community hospitals was perceived to be better than at tertiary care/referral hospitals.

**Table 2.** Themes from 29 comparison studies of the learning environment in the health professions.

LE Components - Citation, Nation, Profession	Comparison Groups	Findings (+, =, -)
Personal		
Carlson (2014) ( Carlson and Idvall, 2014) Sweden Nursing	Prior experience working in elder care vs. no prior experience working in nursing homes	(=) Students with and without prior experience with elder care perceived the nursing home LE similarly. The overall LE in nursing homes rated highly and the supervisory relationship had the highest impact on perceptions of LE.
Social		
Buxton (2014) ( Buxton and De Muth, 2013) USAPharmacy	Live local continuing education program vs. distance webcast program	(=) Both groups were satisfied with what they learned but local group was more satisfied with the learning experience.
Elison-Bowers (2008) ( Elison-Bowers *et al.*, 2008) USACollege students	On-site, remote-site vs. traditional college student perceptions of LE	(=) No differences among groups in any of the four domains of student/teacher interactions, course structure, physical LE and overall satisfaction with course. Students tended to prefer traditional classroom environment.
Makhdoom (2013) ( Makhdoom *et al.*, 2013) Saudi Arabia Medicine	Face-to-face instruction vs. blended learning (electronic and face-to-face)	(+) Blended learning was perceived to be better than traditional learning in all domains of the LE, except for social interactions, and in all types of examinations.
Organizational		
Auret (2013) ( Auret *et al.*, 2013) Australia Medicine	Metropolitan vs rural/ remote clinical placements for residents	(+) Teaching, learner autonomy and support all rated highly in the interns’ responses and the rural rotations scored higher in teaching and support when compared with urban rotations.
Bennett (2010) ( Bennett, Kelly and O’Flynn, 2010) UKMedicine	Tertiary referral hospitals vs. smaller hospitals	(+) Year 3 students’ perceptions of atmosphere, teaching and learning were higher at smaller sites.
Bisholt (2014) ( Bisholt *et al.*, 2014) Sweden Nursing	Hospital department, community-based care, primary care, psychiatric care settings	(+) Nursing students rated LE highest in hospital departments; lowest in psychiatric care settings
Boor (2008) ( Boor *et al.*, 2008) The Netherlands Medicine	Highest vs. lowest scoring OB/GYN departments on LE	(+) Differences identified across departments in student perceptions of LE. Characteristics of departments (legitimacy, clerkship arrangements and focus on personal development) and of students (initial initiatives, continuing development and clerkship fatigue) were major themes. The amount and nature of participation played a central role in all themes.
Condon (2017) ( Condon *et al.*, 2017)Australia Medicine	Large metropolitan hospitals vs. smaller rural hospitals	(+) Greatest satisfaction with the LE and highest examination scores were associated with rural clinical sites, and small cohorts of students from single school.
Conner (2016) ( Conner, Behar-Horenstein and Su, 2016) USA Veterinary Medicine	Required academic hospital veterinary emergency and critical care rotation vs. an elective community hospital emergency and critical care rotation	(+) Students preferred the elective emergency rotation where they had more hands-on experience seeing emergencies with ample opportunities to practice client communication and common emergency procedures.
Denz-Penhey (2010) ( Denz-Penhey and Murdoch, 2010) Australia Medicine	Larger vs. smaller rural and remote longitudinal integrated clinical clerkship sites	(=) No differences in perceptions of LE between large and small remote sites; ratings higher than metropolitan sites.
Edgren (2010) ( Edgren *et al.*, 2010) Sweden Medicine	Two different stages in curriculum reform, moving more toward a student-centered curriculum	(=) LE remained high during the change process although students perceived the lack of a support system for stressed students and the lack of feedback and constructive criticism from teachers.
Finn (2014) ( Finn, Avalos and Dunne, 2014) Ireland Medicine	Traditional discipline-based vs. new systems-based, student-centered, integrated curriculum	(+) Greater satisfaction with LE in new curriculum; students perceived better opportunities to develop interpersonal skills, ask questions and learn about empathy.
Henderson (2006) ( Henderson *et al.*, 2006) Australia Nursing	Three supervisory models: traditional facilitation, individual preceptor and clinical education unit (CEU)	(+) Greatest satisfaction with the preceptor model (because strong, supportive relationships can develop); least with facilitation model; CEU model most sustainable model.
Kaufman (1996) ( Kaufman and Mann, 1996) Canada Medicine	Traditional discipline-based curriculum vs. problem-based curriculum	(+) Students perceived their pre-clinical LE more positively in PBL curriculum than traditional, especially for subscales on enthusiasm and democratic decision-making but were less positive about student-interactions.
Kelly (2012) ( Kelly, Bennett and O’Flynn, 2012) Ireland Medicine	Hospital vs. general practice placements for clerkship students	(+) General practice attachments rated higher than hospital attachments in overall LE.
Moore-West (1986) ( Moore-West *et al.*, 1988) USA Medicine	Primary Care Curriculum (PCC) vs. traditional curriculum	(+/-) Student perceptions of distress in the first two years were less in PCC than traditional curriculum. Students from both curricula perceived the emotional climate and interpersonal relationships among students progressively declined over time, although PCC student perceptions were more positive throughout.
Payne (2013) ( Payne, 2013) USA Nursing	Traditional vs. accelerated second degree BSN programs	(=) No differences in perceptions of the educational environment overall.
Prunuske (2013) ( Prunuske and Deci, 2013) USA Medicine	Student placements in ambulatory sites with and without residents	(=) Clerkship sites with and without residents provide comparable learning experiences and precepting. Students placed in resident training sites appear overwhelmed with diverse opportunities and less support than non-resident sites.
Reed (2011) ( D A Reed *et al.*, 2011) USA Medicine	Pass-fail vs. graded evaluation systems among preclinical medical students	(-) Students in schools using grades had higher levels of stress, emotional exhaustion and depersonalization, were more likely to have burnout, and to have seriously considered dropping out of school than students in schools with pass-fail grading.
Schauber (2015) ( Schauber *et al.*, 2015) Germany Medicine	Traditional vs. problem-based curriculum	(+) PBL curriculum associated with higher ratings of LE than traditional curriculum. Self-regulatory processes and collaborative learning play crucial roles in students’ acquisition of knowledge and perceptions of support regardless of curricular context.
Silkins (2017) ( Silkens *et al.*, 2016) The Netherlands Medicine	Comparison of clinical departments by LE groups as perceived by residents: substandard, adequate, good and excellent performers	(+) Teaching status of the hospital, departments’ average teaching performance, and percentage of time spent on educational activities by faculty predicted departments’ LE performance as perceived by residents.
Smith (2016) ( Smith *et al.*, 2016) USA Medicine	Learning communities vs. no learning communities	(+) Medical schools with learning communities were associated with more positive student perceptions of the schools’ LE compared with schools without learning communities.
Tackett (2015) ( Tackett *et al.*, 2015) USA and Malaysia Medicine	Comparison of LE of single curriculum taught at two different schools	(+) Medical students at the end of their first year rated the LE even more positively in Malaysia than in USA partner school.
Taguchi (2008) ( Taguchi, Ogawa and Sasahara, 2008) Japan Dentistry	Main dental teaching hospital vs. cooperating community dental hospital	(+) Trainees rated LE higher in cooperating community dental hospital than main teaching hospital.
Widyandana (2011) ( Widyandana, Majoor and Scherpbier, 2011) Indonesia Medicine	Comparison of three clinical settings to learn pre-clinical clinical skills: primary health care, secondary health care and tertiary health care	(+) Clerkship students rated the LE highest for learning pre-clinical clinical skills in primary health care settings.
Zawawi (2012) ( Zawawi and Elzubeir, 2012) Saudi Arabia Medicine	Traditional discipline-based curriculum vs. hybrid problem-based curriculum	(+) Students in the PBL curriculum perceived the LE more positively than students in the traditional curriculum.
Physical and Virtual Spaces		
Cannon (2008) ( Cannon *et al.*, 2008) USA Medicine	Medical student vs. resident satisfaction with Veterans Affairs (VA) training	(+) Student overall satisfaction higher than residents with VA training although students’ satisfaction declined over time while residents increased. The LE domain (as opposed to clinical faculty, working environment, physical environment) had the strongest association with overall satisfaction in both groups.
Diwadkar (2010) ( Diwadkar and Jelovsek, 2010) USA Medicine	Junior (years 1 & 2) vs. senior (years 3 & 4) OBGYN resident perceptions the operating room LE in tertiary, regional and community hospitals -	(-) Overall LE, learning opportunities and workload/support subscale scores, rated lower by junior compared with senior residents; tertiary referral hospital rated lower than community and regional hospitals.

### Association Studies

We found 14 studies that reported associations of another important variable (such as burnout, career choice, department academic support) with the LE. These studies included 7 in the personal component, 2 in the social component, 5 in the organizational component, and none in the physical/virtual component ( Table 3). In the personal component, resident performance on their certifying exams was positively associated with perceptions of the LE. Similarly, nursing student effort and grade point averages were also positively related to perceptions of LE. Student well-being was positively associated with having a community of peers, good quality of life and less emotional exhaustion and depersonalization. Students with higher resilience levels had better quality of life and better perceptions of the LE. Resident worries about future endurance/capacity predicted exhaustion and lower ratings of the LE.

In the social component, department educational leadership skills were not related to ratings of the LE. In the organizational component, when clerkships were sorted into provision of high and low supervision of students, students perceived that low supervision clerkship sites offered too few opportunities to examine patients independently, insufficient supervision/no feedback, staff lacked motivation to teach and held negative attitudes towards students, the site had too many students, and there was a lack of organization. Residents perceiving adequate support to succeed had less burnout, better resilience, better job satisfaction, better organizational support, and were more likely to have high performance on the in-service exam. Compliance with common program requirements in residency training was associated with better resident perceptions of the LE.

**Table 3.** Themes from 14 association studies of the learning environment in the health professions.

LE Components - Citation, Nation, Profession	Primary Variables	Findings (+, =, -)
Personal		
Baramee (2003) ( Baramee and Blegen, 2003) Thailand Nursing	Student effort, GPA, hardiness, perceptions of clinical competence and LE of recent graduates	(+) Student effort, perception of clinical LE and program grade point average had direct effects on perceptions of competence whereas hardiness had an indirect effect.
Chinthamitr (2014) ( Chinthammitr and Chierakul, 2014) Thailand Medicine	Resident achievement	(+) Knowledge acquisition among internal medicine residents as determined by board certifying examination was associated with perceptions of a constructive LE, especially satisfaction with program training structure.
Dahlin (2010) ( Dahlin, Fjell and Runeson, 2010) Sweden Medicine	Exhaustion (core to burnout) of first year residents; gender	(-) Resident worries about future endurance/capacity predicted exhaustion, but not performance-based self-esteem. Women’s higher exhaustion scores were explained by their higher worries about future endurance/capacity. LE negatively associated with exhaustion.
Mahendran (2015) ( Mahendran *et al.*, 2015) Singapore Medicine	Career choice; attitudes toward psychiatry	(+) Improvements in attitudes toward psychiatry were correlated with LE when it was perceived to provide inspiration, and enabled students to recognize the merits of psychiatry and effectiveness of treatment although stigma of psychiatry continues.
Skochalek (2016) ( Skochelak *et al.*, 2016) USA Medicine	Student demographic variables; student attributes	(+) At end of first year, students’ perceptions of LE differed across medical schools. Medical school explained 15.6% of variance while student attributes and demographic characteristics accounted for only 2.2% of variance on LE scores.
Tempski (2015) ( Tempski *et al.*, 2015) Brazil Medicine	High vs. low resilience levels of students (the capacity to face and overcome adversities, with personal transformation and growth)	(+) Medical students with higher resilience levels had better quality of life and better perceptions of the educational environment.
Yung (1997) ( Yung, 1997) China Nursing	Ethical decision-making of nursing students; degree vs. certificate students	(+/=) LE was correlated with ethical decision-making in degree students. No differences in perception of LE between two groups.
Social		
Tackett (2017) ( Tackett *et al.*, 2017) Israel, Malaysia, China Medicine	Student well-being; empathy	(+) Favorable overall LE perceptions and a community of peers were associated with good quality of life, and less emotional exhaustion and depersonalization.
Malling (2010) ( Malling *et al.*, 2010) Denmark Medicine	Leadership skills of clinical consultants responsible for resident education	(=) No relationship between the LE in clinical departments and the leadership performance of the educational leaders.
Organizational		
Cross (2006) ( Cross *et al.*, 2006) UK Medicine	Recruitment and retention of specialists	(+) Specialists identified ongoing struggles with different models of workplace learning in postgraduate education: effects of curriculum structure (survival vs. ownership), nature of learning relationships (dependence vs. empowerment through collaboration), approach to assessment of learning (convergent vs. divergent) and prevailing learning climate (service-led expediency vs. personal growth).
De Oliveira Filho (2005) ( De Oliveira Filho, Sturm and Sartorato, 2005) Brazil Medicine	Compliance with common program requirements (CPRs) for residency training	(-) Violations of Brazil’s residency program CPRs were associated with residents’ worse perceptions of general quality of life, quality of life in residency and the LE.
Dolmans (2008) ( Dolmans *et al.*, 2008) The Netherlands Medicine	Clerkships rated highly vs. poorly on supervision	(-) Students perceived that poor clerkship sites offered too few opportunities to examine patients independently, offered insufficient supervision/no feedback, staff lacked motivation to teach and held negative attitudes towards students, the site had too many students, and there was a lack of organization.
Gruppen (2015) ( Gruppen *et al.*, 2015) USA Medicine	Institution vs. specialty influence on resident ratings of LE and workload	(+) Institution had greater influence than specialty on resident perceptions of LE and workload.
Lee (2017) ( Lee *et al.*, 2017) USA Medicine	High vs. low academic resource support (e.g., book stipends, formal in-service review questions, remediation, on-site board prep)	(+) Residents perceiving adequate support to succeed had less burnout, better resilience, better job satisfaction, better organizational support, and were more likely to have high performance on the in-service exam.
Physical and Virtual Spaces		
No studies		

### Themes from Descriptive Studies

While the vast majority of studies of the LE were quantitative and used standardized measures of the LE, a few descriptive studies used qualitative research methods to explore learners’ perceptions of the LE. We found nine descriptive studies that addressed all four components ( Table 4). Student perceptions of a constructive LE were associated with resilience, a focus on personal growth, feeling that they were learning in a meaningful place and becoming part of a community, and that they trusted the system to support them. In the social component, students described constructive LEs as being welcoming with scaffolding relationships and a strong teaching culture. Preceptors were perceived to enjoy teaching and provided appropriate instruction, feedback and role modeling. A poor social environment was characterized by mistreatment, neglect and negative attitudes toward learners, unclear expectations, insufficient supervision and too few opportunities to examine patients independently. In the organizational component, the teaching arrangements were well organized, and there was continuity of participants. Smaller and more rural clinical sites were perceived to be better as was a PBL curriculum. Destructive organizational attributes included lack of clear expectations for learners, failure to integrate students into teams, too many students, and lack of organization. In the physical/virtual component, availability of adequate space for students to interview patients was identified.

**Table 4.** Themes from 9 descriptive studies of interventions in the learning environment in the health professions

LE Components	Themes from descriptive studies of the learning environment(+, =, -)
Personal	(+} Resilience ( Seltz *et al.*, 2016) (+) Personal Growth ( Palmgren and Bolander, 2015) (+) A “meaningful” place ( Palmgren and Bolander, 2015) (+) Being part of a community ( Palmgren and Bolander, 2015) (+) Trust in a regulated system to support them ( Palmgren and Bolander, 2015)
Social	(+) Staff welcoming of learners ( Thomson *et al.*, 2014) (+) Scaffoldingrelationships ( Palmgren and Bolander, 2015) (+) There is a strong teaching culture ( Thomson *et al.*, 2014) (+) Preceptors enjoy teaching ( Thomson *et al.*, 2014) and invest time in doing so ( Wear and Skillicorn, 2009) (+) Teachers role model skills ( Thomson *et al.*, 2014) and values ( Wear and Skillicorn, 2009), observe and give feedback to learners for improvement ( Thomson *et al.*, 2014; Suksudaj *et al.*, 2015), provide clear expectations for learning ( Thomson *et al.*, 2014) (+) Multiple levels of learners together ( Thomson *et al.*, 2014) (-) Mistreatment, neglect of learners, negative attitudes toward learners, unclear expectations for learners ( Castillo-Angeles *et al.*, 2017) (-) Insufficient supervision/no feedback ( Thomson *et al.*, 2014), too few opportunities to examine patients independently ( Thomson *et al.*, 2014), staff unmotivated to teach and held negative attitudes toward students ( Thomson *et al.*, 2014)
Organizational	(+) Teaching arrangements well organized ( Thomson *et al.*, 2014) (+) Continuity of participants (teachers, learners, patients) ( Seltz *et al.*, 2016) (+) Smaller, rural clinical sites perceived as better ( Condon *et al.*, 2017) (+) PBL perceived as less stressful and more meaningful than traditional curriculum ( Moore-West *et al.*, 1988) (-) Unclear expectations of learners ( Thomson *et al.*, 2014), failure to integrate students into surgical teams ( Castillo-Angeles *et al.*, 2017), too many students ( Dolmans *et al.*, 2008), lack of organization ( Dolmans *et al.*, 2008)
Physical and Virtual Spaces	(+) Learning spaces are available ( Seltz *et al.*, 2016)

## Discussion

The vast majority of studies included in this scoping review reported on interventions and influences that had a positive impact on the LE in 18 different countries representing medicine, nursing, dentistry, pharmacy, and veterinary medicine. All four types of studies (interventional, group comparisons, associations with another key variable, and descriptive) described influences on one or more components of the LE. The majority of studies were focused on the organizational component, followed by the social component and the personal component. Very few studies examined the impact of the physical or virtual space component.

Our scoping review sought to answer three research questions, the first of which was: What interventions affect the LE in the health professions? A synthesis of the reported interventions aimed at influencing the LE are reported in Table 5. There were seven classes of influences on the LE (accreditation regulations, curricular interventions, faculty/staff development, grading practices, instructional interventions, placements, physical and virtual spaces, and support services) and 20 specific targets for possible interventions. Since the strength of the interventions displayed in Table 5 were not assesses, the list should be viewed as potential opportunities for improving the LE.

**Table 5.** Selected targets for possible interventions to improve learning environments derived from 68 reviewed studies in the health professions.

Class of influence	Possible Interventions	Supporting Studies
Accreditation Regulations	• Structure of work hours and intensity • Focus on well-being	Lachance (2014) ( Lachance *et al.*, 2014), Schumacher (2014) ( Schumacher *et al.*, 2014), Tackett (2017) ( Tackett *et al.*, 2017), De Oliveira Filho (2005) ( De Oliveira Filho, Sturm and Sartorato, 2005), Dolmans (2008) ( Dolmans *et al.*, 2008)
Curricular Interventions	• Include content on well-being, adaptability, preparation for transitions, clarity of expectations and roles • Create continuity of experience	Sundler (2014) ( Sundler *et al.*, 2014), Hunter (2004) ( Hunter *et al.*, 2004), Conner (2016) ( Conner, Behar-Horenstein and Su, 2016), Edgren (2010) ( Edgren *et al.*, 2010), Finn (2014) ( Finn, Avalos and Dunne, 2014), Kaufman (1996) ( Kaufman and Mann, 1996), Moore-West (1986) ( Moore-West *et al.*, 1988), Schauber (2015) ( Schauber *et al.*, 2015), Zawawi (2012) ( Zawawi and Elzubeir, 2012), Cottingham (2008) ( Cottingham *et al.*, 2008),
Faculty/Staff Development	• Conduct faculty/staff development workshops on learning climate, setting expectations, providing feedback, promoting well-being, serving as a positive role model, preparing for teamwork	Edafe (2013) ( Edafe, Mistry and Chan, 2013), Henderson (2010) ( Henderson *et al.*, 2010), Moystad (2014) ( Moystad *et al.*, 2015), Rubak (2008) ( Rubak *et al.*, 2008), Spickard (1996) ( Spickard, Corbett Jr and Schorling, 1996), Wallin (2015) ( Wallin *et al.*, 2015),
Grading Practices	• Implement pass/fail grading system	Reed (2011) ( D A Reed *et al.*, 2011)
Instructional Interventions	• Establish positive interpersonal relationships and welcoming environment • Create a community of peers and peer coaching/teaching programs • Offer adequate supervision and feedbackEnsure support in times of transition • Emphasize meaning in work • Support emerging autonomy • Communicate clear expectations for learning and performance • Utilize blended learning methods • Eliminate mistreatment and disrespect	Lau (2017) ( Lau *et al.*, 2017), Moutier (2016) ( Moutier *et al.*, 2016), Wallin (2015) ( Wallin *et al.*, 2015), Carlson (2014) ( Carlson and Idvall, 2014), Buxton (2014) ( Buxton and De Muth, 2013), Makhdoom (2013) ( Makhdoom *et al.*, 2013), Smith (2016) ( Smith *et al.*, 2016),
Placements, Physical and Virtual Space	• Create longitudinal placements • Consider rural, community placements • Provide adequate physical space for learning and patient care • Offer adequate on-line learning resources and virtual learning spaces	Buxton (2014) ( Buxton and De Muth, 2013), Elison-Bowers (2008) ( Elison-Bowers *et al.*, 2008), Auret (2013) ( Auret *et al.*, 2013), Bennett (2010) ( Bennett, Kelly and O’Flynn, 2010), Bisholt (2014) ( Bisholt *et al.*, 2014), Condon (2017) ( Condon *et al.*, 2017), Denz-Penhey (2010) ( Denz-Penhey and Murdoch, 2010), Kelly (2012) ( Kelly, Bennett and O’Flynn, 2012), Silkins (2017) ( Silkens *et al.*, 2016), Taguchi (2008) ( Taguchi, Ogawa and Sasahara, 2008), Widyandana (2011) ( Widyandana, Majoor and Scherpbier, 2011), Cannon (2008) ( Cannon *et al.*, 2008), Diwadkar (2010) ( Diwadkar and Jelovsek, 2010), Gruppen (2015) ( Gruppen *et al.*, 2015)
Support Services	• Create coaching, mentoring, peer support programs to sustain personal well-being, adaptability and resilience	Van Hell (2009) ( Van Hell, Kuks and Cohen-Schotanus, 2009), Nishioka (2014) ( Nishioka *et al.*, 2014), Smith (2016) ( Smith *et al.*, 2016), Dahlin (2010) ( Dahlin, Fjell and Runeson, 2010), Tackett (2017) ( Tackett *et al.*, 2017), Tempski (2015) ( Tempski *et al.*, 2015), Lee (2017) ( Lee *et al.*, 2017),

The second and third research questions were:

• ·What components of the LE are targeted by these interventions? Which are ignored?
• What are the theoretical and practice gaps that require additional research on the LE and its dynamics?

These two questions are addressed in relation to each of the four components of the LE.

### Personal Component of LE

The personal component of our LE model describes how individual learners interact with the LE, develop perceptions of the LE, engage in personal growth and develop professional identity. It describes the psychological, experiential and perceptual dimensions of a particular setting. Interventions or factors positively associated with the personal component of LE included: time focused on direct patient care, having a community of peers, a good quality of life and high levels of resilience, learning in a “meaningful” place, and trust in a regulated system to support them. Factors with negative associations were poor quality of life leading to more emotional exhaustion, depersonalization, and worries about future endurance and capacity. These factors are less about interventions and more about the psychological characteristics of the learners and their perceptions of the environment.

Sociocultural learning theories associated with situated learning, situated cognition, ecological psychology, workplace learning explain these findings ( Brown *et al.*, 1989; Lave and Wenger, 1991; Billett, 2001). A supportive learning community encourages participation and scaffolds learning in the context of the setting. Motivation theory, which emphasizes autonomy, purpose/goals, mastery and relatedness also connect with these recommendations ( Deci, Koestner and Ryan, 1999; Pintrich, 2003). Learners are intrinsically motivated to learn, develop autonomy, pursue a goal and purpose larger than themselves, and work collaboratively with others, especially if they are supported in the process.

### Social Component of LE

Studies exploring the social component of learning reinforced the importance of interpersonal relationships in fostering a constructive LE. These relationships include teacher and learner (e.g. face-to-face or blended instruction and longitudinal clinical mentoring), learner to learner (e.g. peer instruction and support), as well as faculty to faculty (e.g. leadership performance). Studies did not address the learner and patient relationship. These studies also underpinned the importance of longitudinal relationships as well as the value of setting and revisiting expectations about performance and relationships. The descriptive studies highlighted the role of a strong teaching culture, strong role model skills and values, multiple levels of learners working together (e.g. near peer teaching) as well as the need to avoid mistreatment, unclear expectations, and insufficient supervision without feedback. Teamwork and its relationship to LE were not explicitly addressed in the studies included in our review. These findings are consistent with situated learning (communities of practice and legitimate peripheral participation), situated cognition, and deliberate practice theory, as noted above.

### Organizational Component of LE

The organizational component of the LE model was most frequently studied through comparative studies of contrasting LEs. Frequently, these contrasting environments were “natural experiments” rather than carefully designed studies specifically of the impact on the LE. Many of these were comparisons of alternative curricular models (e.g., problem-based learning, team-based learning) or specific curricular interventions (e.g., augmenting feedback, faculty development, team-work skills) or larger setting of school comparisons (rural vs urban, alternative clinical settings within a larger academic institution). The uncontrolled and non-randomized nature of these studies limits the confidence one can place in the results, but the evidence is generally positive in indicating that some environments are perceived as better than others. These include:

• Courses or innovations to augment feedback, increase respect and well-being, and reduce mistreatment
• Faculty development programs focused on aspects of the LE rather than specific teaching skills
• Structural features like duty hour implementation, grading systems, supervisory models, and dedicated educational units
• Rural settings, smaller clinical placements, learning communities, and elective rotations, which may be surrogates for having more attention given to learners.

Given the diversity in study outcomes, disciplines, countries, and focus, it is not surprising that the results are often mixed. There is not a critical mass of studies on any given variable to provide convincing conclusions.

Understanding the dynamics of how organizational features relate to the LE clearly builds on the theories of sociocultural and interpersonal interactions cited in the sections on the personal and social components of our model. However, the organizational component also leads to considerations of institutional and organizational culture that are seldom cited in LE studies. Organizational change ( Kotter, 1995; Bolman and Deal, 2013), leadership models ( Avolio, Walumbwa and Weber, 2009), and systems science ( Miller, 1978)are a few of the conceptual domains that may be relevant and beneficial for better understanding how the LE functions at higher level human systems.

### Physical and Virtual Space Component of LE

The physical/virtual space component of the LE encompasses the physical spaces of educational and practice settings in which learning and practice occur, and the virtual or online learning spaces. We identified three studies, two of which were comparison studies ( Cannon *et al.*, 2008; Diwadkar and Jelovsek, 2010)and one a descriptive study ( Seltz *et al.*, 2016), all of which were conducted in the US. Within these studies, physical components of the LE are peripheral rather than the main focus of the study. For example, in a survey of 125 Veterans’ Affairs hospitals, physical space is one of four investigated subdomains that are associated the LE ( Cannon *et al.*, 2008). This study notes that for residents and medical students the maintenance and cleanliness of hospital facilities impacts the LE.

The lack of identified studies and limited coverage suggests a gap in the health professions literature and opportunities for future research. Health professions education researchers might refer to other fields, such as environmental psychology and higher education, as they have long studied the physical/virtual components of the LE and recognize the impact of space on learning ( Oblinger and Lippincott, 2006). Furthermore, a need for knowledge about physical/virtual components of the LE will become more pronounced as health professions education institutions implement blended learning ( Mehta *et al.*, 2013; Prober and Khan, 2013). Using blended learning approaches, faculty intentionally plan their teaching to engage trainees online and in-person to optimize the affordances of both modalities. While blended learning moves some of the learning out of the physical space and into the ether, it underscores the need for those opportunities in the physical learning space to directly support small group learning. In addition, as interprofessional education and practice increase, new spaces for conferences and huddles in the workplace will be needed. Ambulatory clinic space is also required for medical student practice, especially in the early stages of learning when they are inefficient.

We note that the physical and virtual space component received the least attention of the four components in our organizational framework, especially given the amount of time, energy and financial resources devoted to fundraising campaigns targeting expanded and improved physical spaces and online courses (Association of American Medical Colleges, no date). This lack of coverage may in part reflect the absence of sociocultural theoretical stances, where the location and its interaction with participants is a key element. Indeed, we suspect that clarity on definitional and theoretical stance would lead to more (needed) investigations of this component.

### Recommendations

We have several recommendations that arise from this review:

• There is a significant need for theoretical development to provide a more comprehensive framework for both defining the learning environment and studying its impact on various educational outcomes. The need for better definitional and theoretical clarity became evident early in our review process. This lack of clarity led to challenges in constructing our literature search, as well as in synthesizing our findings. We believe that enhancing the definitional and theoretical clarity of the LE is a critical next step to improve our understanding of interventions, the components to target, and addressing practice gaps.
• Similarly, the over-reliance on learner self-reported perceptions as a measure of the learning environment need to be supplemented by assessment methods that better address other viewpoints and the characteristics of the LE at the group and institutional levels. Reviews of assessment instruments are available and note the lack of consistent theoretical frameworks ( Schönrock-Adema *et al.*, 2012; Colbert-Getz *et al.*, 2014).
• There are a number of gaps that warrant research attention: exploring the patient’s impact on the LE, investigating how interprofessional and intra-professional teams influence the LE as well as the design and testing of interventions that are inclusive of multiple components from our model would be worthy of future investigations. Similarly, potential interventions to improve the LE should carefully consider creating a community of peers, ensuring support especially in times of transition and stress, emphasizing meaning in the work, and supporting personal resilience and autonomy. Physical and virtual spaces as settings for learning are also under-represented in the literature.
• Educational scholars and practitioners must recognize that the contextual, background nature of the LE makes it a construct that may or may not be explicitly identified in individual studies. For example, our search returned only two articles ( Lachance *et al.*, 2014; Schumacher *et al.*, 2014)on resident duty hours as an element of the LE. There are, obviously, many more articles that examine the impact of duty hour changes on educational outcomes, but these are seldom labeled as “learning environment” and were thus missed in our search. Care must be taken to search more broadly in a given LE intervention to include articles that do NOT mention “learning environment”.

### Limitations

A particular challenge of conducting a comprehensive literature search for a construct like the LE, is that it has no uniform definition and is often a background phenomenon rather than an explicit component of a study. This challenge meant crafting a search strategy that was focused on the inclusion of the term “LE” and several synonyms. Despite our best efforts, we may have failed to retrieve all relevant articles on the LE because we did not use the right terms (LE or its synonyms). Additionally, we restricted our search to English language journal articles and thus may have excluded relevant research in non-English languages. Since the review was focused on interventions that impact the learning environment, studies that described the LE or validated a LE instrument were excluded. Some of these may have provided further insights into interventions.

## Conclusions

The context in which people learn clearly has an impact on the learning process and its outcomes. This context includes numerous factors at the personal, social, and organizational levels. It also includes physical and virtual spaces. Because of this scope, discussing all of these factors under the term LE would appear to be a gross over-simplification. We argue that research in this area can only progress if investigators and practitioners become clear and precise about what they mean by LE. Clarity and precision will be facilitated by the development of more detailed theoretical models and congruent assessment tools. For example, the model we have developed from this review would suggest that authors should address the “personal learning environment” as distinct from the “social learning environment,” the “organizational learning environment” or the “physical and virtual learning environments”. Such distinctions are necessary to advance future research on the LE by focusing on a subset of components, variables and/or interventions rather than the enormity of all possible contextual influences. Similarly, because the specific LE in a given study is defined by the educational purpose, actions, and outcomes, further theoretical development of the LE concept must incorporate these foreground educational issues in order to understand the dynamics of the LE “background.”

## Take Home Messages

• There is a significant need for theoretical development to defining the dynamics of the learning environment and studying its impact on various educational outcomes.
• There is an over-reliance on learner self-reported perceptions as a measure of the learning environment. Other assessment methods are needed to better address other viewpoints and characteristics of the LE at the group and institutional levels.
• Additional research attention is needed in such areas as exploring the patient’s impact on the LE, investigating how interprofessional and intra-professional teams influence the LE, creating a community of peers, ensuring support especially in times of transition and stress, emphasizing meaning in the work, and supporting personal resilience and autonomy. Physical and virtual spaces as settings for learning are also under-represented in the literature.
• The contextual, background nature of the LE makes it a construct that may or may not be explicitly identified in individual studies. For example, there are many articles that examine the impact of duty hour changes on educational outcomes, but these are seldom labeled as “learning environment” and were thus missed in our search. Care must be taken to search broadly in a given LE intervention to include articles that do NOT mention “learning environment”.

## Notes On Contributors

Larry Gruppen is Professor, Department of Learning Health Systems at the University of Michigan where he directs an innovative, competency-based Master of Health Professions Education program. His scholarly interests center around clinical reasoning, assessment, faculty development, and research methods.

David Irby is Professor Emeritus, Department of Medicine and Senior Scholar, Center for Faculty Educators, UCSF school of Medicine. His scholarly interests center around faculty development and clinical teaching.

Steven Durning is Professor, Department of Medicine and Pathology and Director, Graduate Programs in Health Professions Education. His scholarly interests center around clinical reasoning, assessment, educational theory, and research methods.

Lauren Maggio is associate professor, Department of Medicine, and the Associate Director of Distributed Learning and Technology in the Graduate Programs in Health Professions Education at the Uniformed Services University of the Health Sciences. Her research interests focus on scholarly communication, knowledge syntheses, and health information use.

## Appendices

### APPENDIX 1 - SEARCHES

#### Database: ERIC via ProQuest interface Date: 10/11/2017

(((AB,TI(“education* climate”) OR AB,TI(“education* environment”) OR AB,TI(“learning climate”) OR AB,TI(“learning environment”) OR AB,TI(“classroom climate”) OR AB,TI(“classroom environment”)) AND (AB,TI(“learning environment survey”) OR AB,TI(“clinical learning environment inventory”) OR AB,TI(CLEI) OR AB,TI(“Dundee Ready Educational Environment”) OR AB,TI(DREEM) OR AB,TI(“Medical Student Learning Environment Survey”) OR AB,TI(MSLES) OR AB,TI(“Postgraduate Hospital Educational Environment Measure”) OR AB,TI(PHEEM) OR AB,TI(“Dutch Residency Educational Climate Test”) OR AB,TI(D-RECT) OR AB,TI(“Surgical Theatre Education Environment Measure”) OR AB,TI(STEEM) OR AB,TI(“Undergraduate Clinical Education Environment Measure”) OR AB,TI(UCEEM) OR SU.EXACT(“Measurement”) OR (AB,TI(educat*) AND AB,TI(measure*)) OR SU.EXACT(“Program Evaluation”) OR AB,TI(“program evaluation”) OR ((AB,TI(educat*) OR AB,TI(measure*))AND (SU.EXACT(“Attitudes”) OR AB,TI(attitude*)))) AND (AB,TI(residen*) OR AB,TI(intern*) OR AB,TI(clerkship*) OR AB,TI(fellow*) OR ((AB,TI(student*) OR AB,TI(educat*)) AND (AB,TI(medicine) OR AB,TI(medical) OR AB,TI(physician*) OR AB,TI(nurs*) OR AB,TI(dental) OR AB,TI(dentist*) OR AB,TI(pharmacist*) OR AB,TI(pharmacology) OR AB,TI(pharmacy) OR AB,TI(paramedic*) OR AB,TI(public health) OR AB,TI(premedical) OR AB,TI(allied health))) OR ( **SU.EXACT.EXPLODE(“Medical Education”) AND SU.EXACT.EXPLODE(“Students”)**) OR SU.EXACT(“Professional Education”))) NOT (DTYPE(Opinion) OR (DTYPE(Review) NOT systematic)))

#### Database: Scopus Date: 10/11/2017

(((TITLE-ABS(“education* climate”) OR TITLE-ABS(“education* environment”) OR TITLE-ABS(“learning climate”) OR TITLE-ABS(“learning environment”) OR TITLE-ABS(“classroom climate”) OR TITLE-ABS(“classroom environment”)) AND (TITLE-ABS(“learning environment survey”) OR TITLE-ABS(“clinical learning environment inventory”) OR TITLE-ABS(CLEI) OR TITLE-ABS(“Dundee Ready Educational Environment”) OR TITLE-ABS(DREEM) OR TITLE-ABS(“Medical Student Learning Environment Survey”) OR TITLE-ABS(MSLES) OR TITLE-ABS(“Postgraduate Hospital Educational Environment Measure”) OR TITLE-ABS(PHEEM) OR TITLE-ABS(“Dutch Residency Educational Climate Test”) OR TITLE-ABS(D-RECT) OR TITLE-ABS(“Surgical Theatre Education Environment Measure”) OR TITLE-ABS(STEEM) OR TITLE-ABS(“Undergraduate Clinical Education Environment Measure”) OR TITLE-ABS(UCEEM) OR (TITLE-ABS(educat*) AND TITLE-ABS(measure*)) OR TITLE-ABS(“program evaluation”) OR ((TITLE-ABS(educat*) OR TITLE-ABS(measure*)) AND TITLE-ABS(attitude*))) AND (TITLE-ABS(residen*) OR TITLE-ABS(intern*) OR TITLE-ABS(clerkship*) OR TITLE-ABS(fellow*) OR ((TITLE-ABS(student*) OR TITLE-ABS(educat*)) AND (TITLE-ABS(medicine) OR TITLE-ABS(medical) OR TITLE-ABS(physician*) OR TITLE-ABS(nurs*) OR TITLE-ABS(dental) OR TITLE-ABS(dentist*) OR TITLE-ABS(pharmacist*) OR TITLE-ABS(pharmacology) OR TITLE-ABS(pharmacy) OR TITLE-ABS(paramedic*) OR TITLE-ABS(public health) OR TITLE-ABS(premedical) OR TITLE-ABS(allied health))) OR TITLE-ABS(“Professional Education”))) AND NOT (DOCTYPE(ed) OR DOCTYPE(no) OR DOCTYPE(le) OR DOCTYPE(pr) OR (DOCTYPE(re) AND NOT systematic)))

#### Database: CINAHL Complete Date: 10/11/2017

(((AB “education* climate” OR TI “education* climate” OR AB “education* environment” OR TI “education* environment” OR AB “learning climate” OR TI “learning climate” OR AB “learning environment” OR TI “learning environment” OR AB “classroom climate” OR TI “classroom climate” OR AB “classroom environment” OR TI “classroom environment”) AND (AB “learning environment survey” OR TI “learning environment survey” OR AB “clinical learning environment inventory” OR TI “clinical learning environment inventory” OR AB “CLEI” OR TI “CLEI” OR AB “Dundee Ready Educational Environment” OR TI “Dundee Ready Educational Environment” OR AB “DREEM” OR TI “DREEM” OR AB “Medical Student Learning Environment Survey” OR TI “Medical Student Learning Environment Survey” OR AB “MSLES” OR TI “MSLES” OR AB “Postgraduate Hospital Educational Environment Measure” OR TI “Postgraduate Hospital Educational Environment Measure” OR AB “PHEEM” OR TI “PHEEM” OR AB “Dutch Residency Educational Climate Test” OR TI “Dutch Residency Educational Climate Test” OR AB “D-RECT” OR TI “D-RECT” OR AB “Surgical Theatre Education Environment Measure” OR TI “Surgical Theatre Education Environment Measure” OR AB “STEEM” OR TI “STEEM” OR AB “Undergraduate Clinical Education Environment Measure” OR TI “Undergraduate Clinical Education Environment Measure” OR AB “UCEEM” OR TI “UCEEM” OR (MH “Educational Measurement”) OR (MH “Program Evaluation”) OR AB “program evaluation” OR TI “program evaluation” OR ((AB educat* OR TI educat* OR AB measure* OR TI measure*) AND ((MH “Consumer Attitudes”) OR (MH “Attitude of Health Personnel”) OR AB attitude* OR TI attitude*))) AND (AB residen* OR TI residen* OR AB intern* OR TI intern* OR AB clerkship* OR TI clerkship* OR AB fellow* OR TI fellow* OR ((AB student* OR TI student* OR AB educat* OR TI educat*) AND (AB medicine OR TI medicine OR AB medical OR TI medical OR AB physician* OR TI physician* OR AB nurs* OR TI nurs* OR AB dental TI dental OR AB dentist* TI dentist* OR AB pharmacist* OR TI pharmacist* OR AB pharmacology OR TI pharmacology OR AB pharmacy OR TI pharmacy OR AB paramedic* OR TI paramedic* OR (AB public health) OR (TI public health) OR AB premedical OR TI premedical OR (AB allied health) OR (TI allied health))) OR (MH “Students, Health Occupations”)OR (MH “Education, Health Sciences”))) NOT (ZT “editorial” OR ZT “commentary” OR ZT “letter” OR (ZT “review” NOT ((ZT “systematic review”) OR systematic))))

#### Database: Medline via PubMed interface Date 10/11/2017

(((“education climate”[tiab] OR “educational climate”[tiab] OR “education environment”[tiab] OR “educational environment”[tiab] OR “learning climate”[tiab] OR “learning environment”[tiab] OR “classroom climate”[tiab] OR “classroom environment”[tiab] OR (“distance education”[tiab] AND (environment[tiab] OR climate[tiab]))) AND (“learning environment survey”[tiab] OR “clinical learning environment inventory”[tiab] OR CLEI[tiab] OR “Dundee Ready Educational Environment”[tiab] OR DREEM[tiab] OR “Medical Student Learning Environment Survey”[tiab] OR MSLES[tiab] OR “Postgraduate Hospital Educational Environment Measure”[tiab] OR PHEEM[tiab] OR “Dutch Residency Educational Climate Test”[tiab] OR D-RECT[tiab] OR “Surgical Theatre Education Environment Measure”[tiab] OR STEEM[tiab] OR “Undergraduate Clinical Education Environment Measure”[tiab] OR UCEEM[tiab] OR “Educational Measurement”[Mesh] OR (educat*[tiab] AND measure*[tiab]) OR “Program Evaluation”[Mesh] OR “program evaluation”[tiab] OR ((educat*[tiab] OR measure[tiab] OR measurement[tiab] OR intervention[tiab] OR interventions[tiab]) AND (“Consumer Behavior”[Mesh] OR “Attitude of Health Personnel”[Mesh] OR attitude*[tiab] OR unequal[tiab] OR inequality[tiab] OR inequity[tiab] OR equity[tiab] OR mistreat*[tiab] OR inclusion[tiab] OR inclusiv*[tiab] OR mistreat*[tiab] OR respectful[tiab] OR respect[tiab] OR inquisitive[tiab] OR bully*[tiab] OR bulli*[tiab] OR harass*[tiab] OR “stereotype threat”[tiab] OR intimidat*[tiab]))) AND ((resident[tiab] NOT (“nursing home”[tiab] OR “skilled nursing facility”[tiab])) OR intern*[tiab] OR clerkship*[tiab] OR fellow*[tiab] OR ((student*[tiab] OR educat*[tiab]) AND (medicine[tiab] OR medical[tiab] OR physician*[tiab] OR nurs*[tiab] OR dental[tiab] OR dentist*[tiab] OR pharmacist*[tiab] OR pharmacology[tiab] OR pharmacy[tiab] OR paramedic*[tiab] OR premedical[tiab] OR allied health[tiab])) OR “Students, Health Occupations”[Mesh] OR “Education, Professional”[Mesh])) NOT (Editorial[ptyp] OR Comment[ptyp] OR News[ptyp] OR Newspaper Article[ptyp] OR Observational Study[ptyp] OR review[ptyp)) AND (English[lang])

#### Database: Embase via Embase.com Date 10/11/2017

((‘educational climate’:ti,ab OR ‘education climate’:ti,ab OR ‘education environment’:ti,ab OR ‘educational environment’:ti,ab OR ‘learning climate’:ti,ab OR ‘learning environment’:ti,ab OR ‘classroom climate’:ti,ab OR ‘classroom environment’:ti,ab OR (‘distance education’:ti,ab AND (climate:ti,ab OR environment:ti,ab))) AND (‘learning environment survey’:ti,ab OR ‘clinical learning environment inventory’:ti,ab OR CLEI:ti,ab OR ‘Dundee Ready Educational Environment’:ti,ab OR DREEM:ti,ab OR ‘Medical Student Learning Environment Survey’:ti,ab OR MSLES:ti,ab OR ‘Postgraduate Hospital Educational Environment Measure’:ti,ab OR PHEEM:ti,ab OR ‘Dutch Residency Educational Climate Test’:ti,ab OR D-RECT:ti,ab OR ‘Surgical Theatre Education Environment Measure’:ti,ab OR STEEM:ti,ab OR ‘Undergraduate Clinical Education Environment Measure’:ti,ab OR UCEEM:ti,ab OR (educat*:ti,ab AND measure*:ti,ab) OR ‘program evaluation’/exp OR ‘program evaluation’:ti,ab OR ((educat*:ti,ab OR measure*:ti,ab) AND (‘health personnel attitude’/exp OR attitude*:ti,ab OR ‘consumer attitude’/exp OR unequal:ti,ab OR inequality:ti,ab OR inequity:ti,ab OR equity:ti,ab OR mistreat*:ti,ab OR inclusion:ti,ab OR inclusiv*:ti,ab OR mistreat*:ti,ab OR respectful:ti,ab OR respect:ti,ab OR inquisitive:ti,ab OR bully*:ti,ab OR bulli*:ti,ab OR harass*:ti,ab OR ‘stereotype threat’:ti,ab OR intimidat*:ti,ab))) AND ((residen*:ti,ab NOT (‘nursing home’:ti,ab OR ‘skilled nursing facility’:ti,ab)) OR intern:ti,ab OR clerkship*:ti,ab OR fellow*:ti,ab OR ((student*:ti,ab OR educat*:ti,ab) AND (medicine:ti,ab OR medical:ti,ab OR physician*:ti,ab OR nurs*:ti,ab OR dental:ti,ab OR dentist*:ti,ab OR pharmacist*:ti,ab OR pharmacology:ti,ab OR pharmacy:ti,ab OR paramedic*:ti,ab OR public health:ti,ab OR premedical:ti,ab OR allied health:ti,ab)) OR ‘medical personnel’/exp OR ‘vocational education’/exp) NOT (‘editorial’:it OR ‘note’:it OR ‘letter’:it OR (‘review’:it NOT systematic)))AND [english]/lim

## References

[ref1] Accreditation Council on Graduate Medical Education (no date) Clinical Learning Environment Review overview. Available at: http://www.acgme.org/What-We-Do/Initiatives/Clinical-Learning-Environment-Review-CLER( Accessed: 3 July 2017).

[ref2] Arksey H. and O’Malley L. (2005) Scoping studies: towards a methodological framework. International Journal of Social Research Methodology. 8(1), pp.19–32. 10.1080/1364557032000119616

[ref3] Association of American Medical Colleges (no date) New buildings. Available at: https://www.aamc.org/members/gip/private/149582/newbuildings.html( Accessed: 20 January 2018).

[ref4] Auret K. A. (2013) Formal assessment of the educational environment experienced by interns placed in rural hospitals in Western Australia. Rural and Remote Health. 13(4). 24138301

[ref5] Avolio B. J. Walumbwa F. O. and Weber T. J. (2009) Leadership: current theories, research, and future directions. Annual review of psychology. 60, pp.421–449. 10.1146/annurev.psych.60.110707.163621 18651820

[ref6] Baramee J. and Blegen M. A. (2003) New graduate perception of clinical competence: testing a causal model. Int J Nurs Stud. 2003/04/02,40(4), pp.389–399. 10.1016/S0020-7489(02)00104-9 12667516

[ref7] Bennett D. Kelly M. and O’Flynn S. (2010) Are the bigger hospitals better: DREEM on? Irish Journal of Medical Science. 179(4), pp.515–519. 10.1007/s11845-010-0551-x 20730505

[ref8] Billett S. (2001) Learning through work: workplace affordances and individual engagement. J Workplace Learning. 13(5), pp.209–214. 10.1108/EUM0000000005548

[ref9] Bisholt B. (2014) Nursing students’ assessment of the learning environment in different clinical settings. Nurse Educ Pract. 2013/12/21,14(3), pp.304–310. 10.1016/j.nepr.2013.11.005 24355802

[ref10] Bolman L. G. and Deal T. E. (2013) Reframing Organizations: Artistry, Choice, and Leadership. John Wiley & Sons.

[ref11] Boor K. (2008) How undergraduate clinical learning climates differ: A multi-method case study. Medical Education. Blackwell Publishing Ltd. 42(10), pp.1029–1036. 10.1111/j.1365-2923.2008.03149.x 18823522

[ref12] Brown J. S. (1989) Situated cognition and the culture of learning. Educational Researcher. 18(1), pp.32–42. 10.3102/0013189X018001032

[ref13] Buxton E. C. and De Muth J. E. (2013) Pharmacists’ perceptions of a live continuing education program comparing distance learning versus local learning. Res Social Adm Pharm. 2012/07/28,9(2), pp.230–235. 10.1016/j.sapharm.2012.05.003 22835712

[ref14] Cannon G. W. (2008) Factors determining medical students’ and residents’ satisfaction during VA-based training: Findings from the VA learners’ perceptions survey. Academic Medicine. 83(6), pp.611–620. 10.1097/ACM.0b013e3181722e97 18520472

[ref15] Carlson E. and Idvall E. (2014) Nursing students’ experiences of the clinical learning environment in nursing homes: a questionnaire study using the CLES+T evaluation scale. Nurse education today. 34(7), pp.1130–1134. 10.1016/j.nedt.2014.01.009 24529997

[ref16] Castillo-Angeles M. (2017) Mistreatment and the learning environment for medical students on general surgery clerkship rotations: What do key stakeholders think? American Journal of Surgery. 213(2), pp.307–312. 10.1016/j.amjsurg.2016.10.013 28131325

[ref17] Chinthammitr Y. and Chierakul N. (2014) Learning environment and resident achievement. J Med Assoc Thai. 2015/03/15,97(12), pp.1269–1273. 25764633

[ref18] Colbert-Getz J. M. (2014) Assessing Medical Students’ and Residents’ Perceptions of the Learning Environment: Exploring Validity Evidence for the Interpretation of Scores From Existing Tools. Academic Medicine. 89, pp.1687–1693. 10.1097/ACM.0000000000000433 25054415

[ref19] Condon B. P. (2017) Student academic performance in rural clinical schools: The impact of cohort size and competition. Med Teach. 2016/12/31,39(3), pp.262–268. 10.1080/0142159X.2017.1270430 28033729

[ref20] Conner B. J. Behar-Horenstein L. S. and Su Y. (2016) Comparison of Two Clinical Teaching Models for Veterinary Emergency and Critical Care Instruction. J Vet Med Educ. 2016/01/12,43(1), pp.58–63. 10.3138/jvme.0415-069R1 26751912

[ref21] Cottingham A. H. (2008) Enhancing the informal curriculum of a medical school: a case study in organizational culture change. J Gen Intern Med. 2008/04/05,23(6), pp.715–722. 10.1007/s11606-008-0543-y 18389324 PMC2517875

[ref22] Cross V. (2006) Perceptions of the learning environment in higher specialist training of doctors: implications for recruitment and retention. Medical Education. 2006/02/03. England: School of Health, University of Wolverhampton, 10.1111/j.1365-2929.2005.02382.x 16451239

[ref23] Wolverhampton UK . vinette.cross@wlv.ac.uk. 40(2), pp.121–128. 10.1111/j.1365-2929.2005.02382.x

[ref24] Dahlin M. Fjell J. and Runeson B. (2010) Factors at medical school and work related to exhaustion among physicians in their first postgraduate year. Nordic Journal of Psychiatry. 64(6), pp.402–408. 10.3109/08039481003759219 20429747

[ref25] Deci E. Koestner R. and Ryan R. (1999) A meta-analytic review of experiments examining the effects of extrinsic rewards on intrinsic motivation. Psychological Bulletin. 125(6), pp.627–668. 10.1037/0033-2909.125.6.627 10589297

[ref26] Denz-Penhey H. and Murdoch J. C. (2010) Is small beautiful? Student performance and perceptions of their experience at larger and smaller sites in rural and remote longitudinal integrated clerkships in the Rural Clinical School of Western Australia. Rural Remote Health. 10(3), p.1470. 20858018

[ref27] Diwadkar G. B. and Jelovsek J. E. (2010) Measuring surgical trainee perceptions to assess the operating room educational environment. Journal of Surgical Education. 67(4), pp.210–216. 10.1016/j.jsurg.2010.04.006 20816355

[ref28] Dolmans D. H. J. M. (2008) Factors adversely affecting student learning in the clinical learning environment: A student perspective. Education for Health: Change in Learning and Practice. 21(3). 19967634

[ref29] Edafe O. Mistry N. and Chan P. (2013) First impressions count: does FAIRness affect adaptation of clinical clerks in their first clinical placement? Med Teach. 2013/07/03,35(9), pp.740–746. 10.3109/0142159X.2013.801944 23808587

[ref30] Edgren G. (2010) Comparing the educational environment (as measured by DREEM) at two different stages of curriculum reform. Medical teacher. 32(6), pp.e233–238. 10.3109/01421591003706282 20515368

[ref31] Elison-Bowers P. (2008) Health science students and their learning environment: a comparison of perceptions of on-site, remote-site, and traditional classroom students. Perspect Health Inf Manag. 2008/03/04,5, p.2. 18311326 PMC2242346

[ref32] Engstrom M. (2017) Nursing students’ perceptions of using the Clinical Education Assessment tool AssCE and their overall perceptions of the clinical learning environment - A cross-sectional correlational study. Nurse Educ Today. 2017/01/29,51, pp.63–67. 10.1016/j.nedt.2017.01.009 28130975

[ref33] Finn Y. Avalos G. and Dunne F. (2014) Positive changes in the medical educational environment following introduction of a new systems-based curriculum: DREEM or reality? Curricular change and the Environment. Irish Journal of Medical Science. 183(2), pp.253–258. 10.1007/s11845-013-1000-4 23943152

[ref34] Genn J. M. (2001a) AMEE Medical Education Guide No. 23 (Part 1): Curriculum, environment, climate, quality and change in medical education - a unifying perspective. Medical Teacher. 23(4), pp.337–344. 10.1080/01421590120063330 12098379

[ref35] Genn J. M. (2001b) AMEE medical education guide no. 23 (Part 2): Curriculum, environment, climate, quality and change in medical education-a unifying perspective. Medical Teacher. 23(4), pp.445–454. 12098364 10.1080/01421590120075661

[ref36] Gruppen L. D. (2015) Institution and Specialty Contribute to Resident Satisfaction With Their Learning Environment and Workload. Academic Medicine. 2015/10/28,90(11 Suppl), pp.S77–82. 10.1097/ACM.0000000000001361 26505106 PMC4624224

[ref37] Gruppen L. D. Rytting M. E. and Marti K. C. (2017) The educational environment.in Dent J. A. Harden R. M. and Hunt D. (eds) A practical guide for medical teachers. 5th edn. Edinburgh: Elsevier, pp.376–383.

[ref38] Gruppen L. D. and Stansfield R. B. (2016) Individual and Institutional Components of the Medical School Educational Environment. Academic Medicine. 91(11), pp.S53–S57. 10.1097/ACM.0000000000001361 27779510

[ref39] Van Hell E. A. Kuks J. B. M. and Cohen-Schotanus J. (2009) Time spent on clerkship activities by students in relation to their perceptions of learning environment quality. Medical Education. 43(7), pp.674–679. 10.1111/j.1365-2923.2009.03393.x 19573191

[ref40] Henderson A. (2006) An evaluation of the first year of a collaborative tertiary-industry curriculum as measured by students’ perception of their clinical learning environment. Nurse Educ Pract. 2008/12/02,6(4), pp.207–213. 10.1016/j.nepr.2006.01.002 19040879

[ref41] Henderson A. (2010) Creating supportive clinical learning environments: an intervention study. J Clin Nurs. 2009/08/19,19(1-2), pp.177–182. 10.1111/j.1365-2702.2009.02841.x 19686319

[ref42] Holt M. C. and Roff S. (2004) Development and validation of the anesthetic theatre educational environment measure (ATEEM). Medical Teacher. 26(6), pp.553–558. 10.1080/01421590410001711599 15763835

[ref43] Hunter A. J. (2004) Medical student evaluation of the quality of hospitalist and nonhospitalist teaching faculty on inpatient medicine rotations. Acad Med. 2003/12/24,79(1), pp.78–82. 10.1097/00001888-200401000-00017 14691002

[ref44] Issenberg B. S. (2005) Features and uses of high-fidelity medical simulations that lead to effective learning: a BEME systematic review. Medical Teacher. 27(1), pp.10–28. 10.1080/01421590500046924 16147767

[ref45] Janz T. A. and Pyke S. A. (2000) A scale to assess student perceptions of academic climates. Canadian Journal of Higher Education. 30(1), pp.89–122.

[ref46] Kaufman D. M. and Mann K. V (1996) Comparing students’ attitudes in problem-based and conventional curricula. Academic Medicine. 71(10), pp.1096–1099. 10.1097/00001888-199610000-00018 9177645

[ref47] Kelly M. Bennett D. and O’Flynn S. (2012) General practice: the DREEM attachment? Comparing the educational environment of hospital and general practice placements. Education for primary care: an official publication of the Association of Course Organisers, National Association of GP Tutors, World Organisation of Family Doctors. 23(1), pp.34–40. 10.1080/14739879.2012.11494068 22306143

[ref48] Kotter J. P. (1995) Leading change: Why transformation efforts fail. Harvard Business Review. (March-April), pp.59–67.

[ref49] Lachance S. (2014) Perceived effects of the 16-hour workday restriction on surgical specialties: Quebec’s experience. Journal of Surgical Education. 71(5), pp.707–715. 10.1016/j.jsurg.2014.01.008 24818538

[ref50] Lau J. N. (2017) A mixed-methods analysis of a novel mistreatment program for the surgery core clerkship. Academic Medicine. 92(7), pp.1028–1034. 10.1097/ACM.0000000000001575 28121657

[ref51] Lave J. and Wenger E. (1991) Situated Learning: Legitimate Peripheral Participation. New York: Cambridge University Press. 10.1017/CBO9780511815355

[ref52] Lee N. (2017) Improving resident well-being and clinical learning environment through academic initiatives. Journal of Surgical Research. 215, pp.6–11. 10.1016/j.jss.2017.02.054 28688662

[ref53] Levac D. Colquhoun H. and O’Brien K. K. (2010) Scoping studies: advancing the methodology. Implementation Science. 5(1), p.69. 10.1186/1748-5908-5-69 20854677 PMC2954944

[ref54] Mahendran R. (2015) The impact of the educational environment on career choice and attitudes toward psychiatry. Medical Teacher. 37(5), pp.494–497. 10.3109/0142159X.2015.1009021 25693795

[ref55] Makhdoom N. (2013) “Blended learning” as an effective teaching and learning strategy in clinical medicine: A comparative cross-sectional university-based study. Journal of Taibah University Medical Sciences. 8(1), pp.12–17. 10.1016/j.jtumed.2013.01.002

[ref56] Malling B. (2010) Educational climate seems unrelated to leadership skills of clinical consultants responsible of postgraduate medical education in clinical departments. BMC Med Educ. 2010/09/23,10, p.62. 10.1186/1472-6920-10-62 20858255 PMC2955595

[ref57] Mehta N. B. (2013) Just imagine: new paradigms for medical education. Academic Medicine. 88(10), pp.1418–1423. 10.1097/ACM.0b013e3182a36a07 23969368

[ref58] Miller J. G. (1978) Living Systems. New York: McGraw-Hill.

[ref59] Moore-West M. (1988) Distress and attitudes towards learning environment: Effects of a curriculum innovation. Teaching & Learning in Medicine. 1(3). 10.1080/10401338909539400 3641579

[ref60] Moos R. H. (1974) The social climate scales: An overview. Palo Alto, CA: Consulting Psychologists Press.

[ref61] Moos R. H. (1980) Evaluating Classroom environments. Studies in Educational Evaluation. 6(1979), pp.239–252.

[ref62] Moutier C. (2016) The Culture of Academic Medicine: Faculty Behaviors Impacting the Learning Environment. Academic psychiatry : the journal of the American Association of Directors of Psychiatric Residency Training and the Association for Academic Psychiatry. 40(6), pp.912–918. 27368643 10.1007/s40596-016-0582-3

[ref63] Moystad A. (2015) Faculty development for clinical teachers in dental education. Eur J Dent Educ. 2014/08/20,19(3), pp.149–155. 25135255 10.1111/eje.12115

[ref64] Nishioka V. M. (2014) Dedicated education unit: student perspectives. Nurs Educ Perspect. 2014/10/09,35(5), pp.301–307. 10.5480/14-1380 25291925

[ref65] Oblinger D. and Lippincott J. K. J. K. (2006) Learning Spaces, Brockport Bookshelf. 78. Available at: https://digitalcommons.brockport.edu/bookshelf/78( Accessed: 20 August 2005).

[ref66] De Oliveira Filho G. R. Sturm E. J. H. and Sartorato A. E. (2005) Compliance with common program requirements in Brazil: Its effects on resident’s perceptions about quality of life and the educational environment. Academic Medicine. 80(1), pp.98–102. 10.1097/00001888-200501000-00023 15618103

[ref67] Palmgren P. and Bolander L. K. (2015) Exploring chiropractic students’ experiences of the educational environment in healthcare professional training: a qualitative study. BMC Medical Education.(15), p.128. 10.1186/s12909-015-0417-z 26242296 PMC4526181

[ref68] Palmgren P. J. (2016) It takes two to tango: An inquiry into healthcare professional education environments. (Doctoral Thesis) Karolinsk Institute, Stockholm. Karolinska Institutet, Stockholm.

[ref69] Payne L. K. (2013) Comparison of students’ perceptions of educational environment in traditional vs. accelerated second degree BSN programs. Nurse Educ Today. 2012/11/22,33(11), pp.1388–1392. 23168141 10.1016/j.nedt.2012.11.003

[ref70] Pintrich P. (2003) A motivational science perspective on the role of student motivation in learning and teaching contexts. Journal of Educational Psychology. 95(4), pp.667–686. 10.1037/0022-0663.95.4.667

[ref71] Prober C. G. and Khan S. (2013) Medical education reimagined: a call to action. Academic Medicine. 88(10), pp.1407–1410. 10.1097/ACM.0b013e3182a368bd 23969367

[ref72] Prunuske J. P. and Deci D. M. (2013) Learning environment: the impact of clerkship location on instructional quality. Family Medicine. United States: Department of Family Medicine and Community Health, University of Minnesota Medical School Duluth, Duluth, MN 55812-3031, USA. jprunusk@d.umn.edu. 45(3), pp.193–196. 23463433

[ref73] Reed D. A. (2011) Relationship of pass/fail grading and curriculum structure with well-being among preclinical medical students: a multi-institutional study. Acad Med. 2011/09/29,86(11), pp.1367–1373. 10.1097/ACM.0b013e3182305d81 21952063

[ref74] Roff S. and McAleer Sean Sue Roff S. (2001) What is educational climate? Medical Teacher. 23(4), pp.333–334. 10.1080/01421590120063312 12098377

[ref75] Rubak S. (2008) A controlled study of the short- and long-term effects of a Train the Trainers course. Med Educ. 2008/05/30,42(7), pp.693–702. 10.1111/j.1365-2923.2008.03044.x 18507769

[ref76] Schauber S. K. (2015) The role of environmental and individual characteristics in the development of student achievement: a comparison between a traditional and a problem-based-learning curriculum. Adv Health Sci Educ Theory Pract. 2015/01/27,20(4), pp.1033–1052. 10.1007/s10459-015-9584-2 25616720

[ref77] Schönrock-Adema J. (2012) Key elements in assessing the educational environment: Where is the theory? Advances in Health Sciences Education. 17, pp.727–742. 10.1007/s10459-011-9346-8 22307806 PMC3490064

[ref78] Schumacher D. J. (2014) The 2011 ACGME standards: impact reported by graduating residents on the working and learning environment. Acad Pediatr. 2014/03/08,14(2), pp.149–154. 10.1016/j.acap.2013.09.002 24602577

[ref79] Seltz L. B. (2016) Ward Rounds With or Without an Attending Physician: How Interns Learn Most Successfully. Academic Pediatrics. 16(7), pp.638–644. 10.1016/j.acap.2016.05.149 27283038

[ref80] Silkens M. E. W. M. (2016) Focus on quality: Investigating residents’ learning climate perceptions. PLoS ONE. 11(1). 10.1371/journal.pone.0147108 PMC4713097 26765742

[ref81] Skochelak S. E. (2016) Medical Student Perceptions of the Learning Environment at the End of the First Year: A 28-Medical School Collaborative. Academic medicine : journal of the Association of American Medical Colleges. 91(9), pp.1257–1262. 26959222 10.1097/ACM.0000000000001137

[ref82] Smith S. D. (2016) Medical Student Perceptions of the Learning Environment: Learning Communities Are Associated With a More Positive Learning Environment in a Multi-Institutional Medical School Study. Academic medicine : journal of the Association of American Medical Colleges. 91(9), pp.1263–1269. 27119332 10.1097/ACM.0000000000001214

[ref83] Spickard I. A. Corbett E. C. Jr and Schorling J. B. (1996) Improving residents’ teaching skills and attitudes toward teaching. Journal of General Internal Medicine. 11(8), pp.475–480. 10.1007/BF02599042 8872785

[ref84] Suksudaj N. (2015) Features of an effective operative dentistry learning environment: students’ perceptions and relationship with performance. Eur J Dent Educ. 2014/05/02,19(1), pp.53–62. 24779719 10.1111/eje.12102

[ref85] Sundler A. J. (2014) Student nurses’ experiences of the clinical learning environment in relation to the organization of supervision: a questionnaire survey. Nurse Educ Today. 2013/07/16,34(4), pp.661–666. 23850574 10.1016/j.nedt.2013.06.023

[ref86] Tackett S. (2015) Learning environment assessments of a single curriculum being taught at two medical schools 10,000 miles apart. BMC Medical Education. BMC Medical Education. 15(1), p.105. 10.1186/s12909-015-0388-0 26081751 PMC4522132

[ref87] Tackett S. (2017) International study of medical school learning environments and their relationship with student well-being and empathy. Medical Education. 51(3), pp.280–289. 10.1111/medu.13120 27896846

[ref88] Taguchi N. Ogawa T. and Sasahara H. (2008) Japanese dental trainees’ perceptions of educational environment in postgraduate training. Medical Teacher. 30(7), pp.e189–e193. 10.1080/01421590802158385 18777418

[ref89] Tempski P. (2015) Relationship among medical student resilience, educational environment and quality of life. PLoS ONE. 10(6). 10.1371/journal.pone.0131535 PMC4486187 26121357

[ref90] Thomson J. S. (2014) The learner’s perspective in GP teaching practices with multi-level learners: a qualitative study. BMC medical education. 14, p.55. 10.1186/1472-6920-14-55 24645670 PMC3995295

[ref91] Wallin C. J. (2015) Creating an environment for patient safety and teamwork training in the operating theatre: A quasi-experimental study. Medical Teacher. 37(3), pp.267–276. 10.3109/0142159X.2014.947927 25180879

[ref92] Wear D. and Skillicorn J. (2009) Hidden in plain sight: the formal, informal, and hidden curricula of a psychiatry clerkship. Acad Med. 2009/03/26,84(4), pp.451–458. 10.1097/ACM.0b013e31819a80b7 19318777

[ref93] Weiss K. B. Wagner R. and Nasca T. J. (2012) Development, Testing, and Implementation of the ACGME Clinical Learning Environment Review (CLER) Program. Journal of graduate medical education. 4(3), pp.396–8. 10.4300/JGME-04-03-31 23997895 PMC3444205

[ref94] Widyandana D. Majoor G. D. and Scherpbier A. J. (2011) Comparison of three clinical environments for pre-clinical clinical skills training. Medical teacher. 33(11), pp.928–932. 10.3109/0142159X.2011.558141 21592019

[ref95] Yung H. H. (1997) Ethical decision-making and the perception of the ward as a learning environment: a comparison between hospital-based and degree nursing students in Hong Kong. Int J Nurs Stud. 1997/04/01,34(2), pp.128–136. 10.1016/S0020-7489(96)00046-6 9134468

[ref96] Zawawi A. H. and Elzubeir M. (2012) Using DREEM to compare graduating students′ perceptions of learning environments at medical schools adopting contrasting educational strategies. Medical Teacher. 34(s1), pp.S25–S31. 10.3109/0142159X.2012.656747 22409187

