# Supplementary material for: Interventions Designed to Improve the Learning Environment in the Health Professions: A Scoping Review
Source: MedEdPublish (2016). 2018 Sep 12;7:211. [Version 1] doi: 10.15694/mep.2018.0000211.1 (PMC10701832; doi:10.15694/mep.2018.0000211.1)
Supplement: Peer review history

# Reviewer report: Reviewer response for version 1

**DOI:** 10.21956/mep.19673.r29380

**Published:** 2019-05-14

**Authors:** Ken Masters

**Affiliations:** 1 Sultan Qaboos University

This review has been migrated. The reviewer awarded 5 stars out of 5 The paper is designed to identify and describe interventions that affect the learning environment in health professions education, the factors that influence the learning environment, and the gaps requiring additional research.This is an extremely comprehensive, detailed and splendid study. The authors begin by explaining the complexities as indicated in the literature, sourced from several disciplines. They use this information to create a clear theoretical background against which to conduct their review. (I’m not sure that Figure 1 adds much to the text already written, but it is a useful visualisation for some readers).Although the authors say that they have not conducted a systematic literature review, the search methodology is described to an impressive level of detail. The presentation of the Results is similarly impressive; the amount of work and attention to detail has resulted in an extremely valuable resource for the reader.One minor point: Figure 2 should probably have been placed immediately after the first paragraph of the Results, as that it where the search results are described, and where such a figure is usually placed.Overall, although the paper is a long read, it is an absolutely outstanding piece of work, and surely stands as an incredibly useful resource. The tables in the Results, by themselves, will surely be used as a useful starting point for readers who wish to engage with issues surrounding the learning environment; I have no hesitation is awarding five stars, and recommending this paper to be read by anyone involved in the organisational development of the medical learning environment.

Reviewer Expertise:

NA

No decision status is available

# Reviewer report: Reviewer response for version 1

**DOI:** 10.21956/mep.19673.r29379

**Published:** 2018-10-26

**Authors:** Richard Hays

**Affiliations:** 1 James Cook University

This review has been migrated. The reviewer awarded 4 stars out of 5 A paper like this is not easy to review. It is a scholarly review of the literature on the topic of 'learning environment' and the results show that many factors are in the mix. The use of the term 'scoping review' may be a little confusing, as there is a lot of debate at the moment about the different kinds of reviews (systematic, scoping, 'best evidence', narrative etc). The method used seemed fine to me but I am not sure whether the 'scoping' narrowed the range of literature searched. The conclusion also asks more questions than it answers. Yes, a learning environment is a complex mix of individual factors, but is any guidance available about how the more important factors may be selected or combined? After reading this paper, I am more confident that I understand the theoretical perspectives, but none the wiser when it comes to working out how to get the chemistry right.

Reviewer Expertise:

NA

No decision status is available
